# Motion‐Style Scalp Acupuncture Ameliorates Post‐Stroke Muscle Spasticity Through Cerebral Blood Flow Augmentation and 5‐HT_2A_R‐Mediated Spinal KCC2 Reactivation

**DOI:** 10.1002/brb3.71064

**Published:** 2025-11-26

**Authors:** Qin‐Yong Zhang, Jun‐Xiang Wang, Liang‐Xiao Ma, Jin‐Shan Zhong, Fei Li, Xu Qian, Ling‐Hui Ma, Jing‐Yun Xiu, Xiu‐Yan Wang

**Affiliations:** ^1^ School of Acupuncture‐Moxibustion and Tuina Beijing University of Chinese Medicine Chaoyang District Beijing China; ^2^ School of Nursing Beijing University of Chinese Medicine Chaoyang District Beijing China; ^3^ The Key Unit of State Administration of Traditional Chinese Medicine Evaluation of Characteristic Acupuncture Therapy Chaoyang District Beijing China

**Keywords:** acupuncture, ischemic stroke, muscle spasticity, spinal motoneuron

## Abstract

**Background:**

Muscle spasticity, a prevalent and debilitating motor impairment after stroke, severely compromises patients’ functional recovery and quality of life. Motion‐style scalp acupuncture (MSSA), a novel integrative therapy combining scalp acupuncture (SA) with targeted exercise training, has emerged as a promising strategy for post‐stroke spasticity (PSS). This study investigated the mechanisms underlying MSSA's therapeutic effects, focusing on 5‐HT_2A_R‐mediated KCC2 reactivation in spinal motoneurons.

**Methods:**

Using a rat model of permanent middle cerebral artery (MCA) occlusion, we evaluated MSSA's effects through behavioral assessments (Zea Longa, modified Ashworth scale (MAS), screen test), cerebral blood flow (laser speckle imaging), and infarct area analysis (TTC staining). Spinal reflex excitability was quantified via H‐reflex electrophysiological recordings, while molecular mechanisms were examined using Western blot, RT‐qPCR, and double immunofluorescence labeling.

**Results:**

Our results demonstrated that MSSA significantly improved neurological function, reduced muscle tone, and attenuated spinal hyperreflexia. These improvements were associated with enhanced cerebral blood flow and reduced infarct areas. Furthermore, MSSA significantly upregulated the expression of 5‐HT_2A_R and KCC2 in the spinal cord. Pharmacological validation using a 5‐HT_2A_R antagonist and agonist confirmed the crucial role of 5‐HT_2A_R in mediating MSSA's therapeutic effects.

**Conclusion:**

These findings demonstrate that MSSA ameliorates PSS through dual mechanisms: improving cerebral blood flow to the ischemic penumbra and restoring spinal inhibitory control via 5‐HT_2A_R‐dependent KCC2 reactivation. This study not only elucidates the mechanisms of MSSA but also provides a novel therapeutic strategy for PSS management.

## Graphical abstract

Pathology of PSS and the antispastic mechanism of MSSA: A focus on cerebral blood flow augmentation and 5‐HT_2A_R‐mediated KCC2 activation. Abbreviations: CST, corticospinal tract; CRT, corticoreticular tract, RST, reticulospinal tract; rCBF, regional cerebral blood flow; PSS, post‐stroke spasticity.

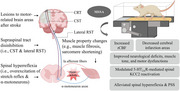

## 1 Introduction

Muscle spasticity, a hallmark motor impairment following stroke, affects 17% to 43% of patients within the first year post‐stroke (Zeng et al. 2021). PSS is clinically defined as a velocity‐ and muscle length‐dependent increase in resistance to externally imposed muscle stretch, which progressively worsens over time. This condition significantly impairs daily life activities and social participation among stroke survivors, while also placing a substantial economic burden on global healthcare systems (Li et al. 2021). Consequently, effective management of PSS has become a critical focus in both medical and nursing fields.

The pathophysiology of PSS is rooted in the neurological disruptions caused by ischemic stroke. The acute deprivation of oxygen and glucose in brain tissues triggers a cascade of events, including excessive release of excitatory neurotransmitters, neuronal death, and axonal degeneration. These processes disrupt communication between supraspinal regions and the spinal cord (Paul et al. 2021). Specifically, the CST loses its direct descending control over spinal motoneurons, leading to CST disinhibition (Huang et al. 2024). Similarly, the lateral RST experiences reduced inhibitory effects on the spinal stretch reflex due to weakened facilitation from the contralateral motor cortex. Concurrently, the medial RST, which operates independently of the contralateral side, exerts an excitatory descending influence, inducing sub threshold discharging of motoneurons and spinal hyperreflexia. These mechanisms collectively contribute to the development of post‐stroke spastic hypertonia (Wang et al. 2023).

At the spinal level, hyperreflexia and decreased inhibitory synaptic transmission are key factors underlying spasticity. Potassium‐chloride cotransporter 2 (KCC2), the primary chloride extruder in mature mammalian neurons, plays a crucial role in maintaining intracellular chloride concentrations below the electrochemical equilibrium potential. This process supports γ‐aminobutyric acid (GABA) A receptor‐mediated neuronal hyperpolarization, thereby alleviating spasticity (Talifu et al. 2022; Bilchak et al. 2021), as demonstrated in our prior research (Wang et al. 2021; Mu et al. 2022; Sun et al. 2022). Serotonin (5‐HT), a key neuromodulator, has garnered significant attention for its role in regulating spinal reflex excitability (Thorstensen et al. 2024; Kavanagh et al., 2022). Emerging evidence suggests that 5‐HT_2A_R receptor activation enhances KCC2 expression on motoneuron membranes, a mechanism associated with the alleviation of spasticity and neuropathic pain following spinal cord injury (Sánchez‐Brualla et al. 2018; Bos et al. 2013).

Acupuncture, recognized as a vital alternative and complementary therapeutic approach, has been endorsed by the World Health Organization (WHO) for stroke rehabilitation and management (Paul et al. 2021; Zhang et al. 2023). Among various acupuncture modalities, MSSA has emerged as a distinctive technique, integrating targeted exercise training during the needle retention period of SA. Clinical evidences have consistently demonstrated that the antispastic effect of MSSA significantly outperforms standalone SA or exercise training in stroke survivors (Zhong et al. 2025; Shi et al. 2024; Liu et al. 2023; Zhang et al. 2022). Our previous experimental work validated that MSSA effectively alleviates PSS in rats by suppressing spinal reflex excitability (Zhang et al. 2023). However, the biomolecular mechanisms underlying MSSA's efficacy in treating PSS remain incompletely understood. Given the established role of 5‐HT_2A_R‐mediated KCC2 activation in chloride homeostasis and spasticity alleviation, we hypothesize that MSSA's antispastic effects may be partially attributed to 5‐HT_2A_R‐mediated KCC2 activation in spinal motoneurons.

## 2 Materials and Methods

### 2.1 Animals and Ethics

One‐hundred and thirty‐two male SD rats (250‐280 g) were purchased from Beijing Vital River Laboratory Animal Technology (License No. SCXK(Jing)2021‐0011). Male rats were chosen for their stable testosterone levels, which minimize interference with neural plasticity and inflammatory responses, consistent incidence of neurological deficits/muscle spasticity in ischemic stroke models to reduce experimental errors, and methodological alignment with prior studies (Shi et al. 2013; Wang et al. 2021) to ensure result comparability. Rats were subjected to one‐week adaptive feeding. All the rats were housed under identical living conditions: constant temperature (23 ± 2°C), standard light/dark cycle, and free access to food and water. The experimental protocol was duly approved by the Animal Ethics Committee of Beijing University of Chinese Medicine (Approval No. BUCM‐4‐2022070401‐3005) and was carried out in strict accordance with the Animal Research: Reporting In Vivo Experiments (ARRIVE) guidelines.

### 2.2 Model Establishment

Rat PSS is primarily driven by cortical injury‐induced spinal excitability abnormalities (Caleo 2015), whereas human spasticity may involve more complex pathological mechanisms, including subcortical structure (e.g., basal ganglia) damage and neuroinflammation (Qin et al. 2022). Nevertheless, evidence has confirmed that stroke‐induced immunodepression in humans not only aligns with rodent models but also shares highly similar phenotypic characteristics (Dirnagl et al. 2007). Previous studies on rat PSS models have shown that muscle tone progressively increases three days after MCA occlusion (MCAO), peaks at day 5, and remains elevated for 10 days (Shi et al. 2013). Considering the model's stability and the defined temporal progression of spastic hypertonia, we established a permanent MCAO model in rats to mimic the disease trajectory and pathological features of human PSS (McBride et al. 2017).

In brief, rats were anesthetized with isoflurane (3 %) in O_2_. The right common carotid artery (CCA), internal carotid artery (ICA), and external carotid artery (ECA) were separated bluntly. A nylon monofilament suture (ɸ 0.36 ± 0.02 mm, Beijing Cinontech Co. Ltd., China) was inserted from the ECA to the ICA, and carefully advanced to block the origin of the MCA, approximately 18–20 mm to the carotid bifurcation. Subsequently, the incisions were sutured in layers. Rats were placed on a heating pad until they were awake. The sham‐operated groups merely underwent separations of the CCA, ECA, and ICA. Ampicillin was given (100 mg/kg, i.p.) for three days to prevent infection. Three days after surgery, an investigator blinded to the group assignment evaluated the neurological deficit of rats with the Zea Longa score and the muscle tone with the MAS. Rats were enrolled with the standards of Zea Longa score and MAS over one.

### 2.3 Acupuncture Treatment

**FIGURE 1:**
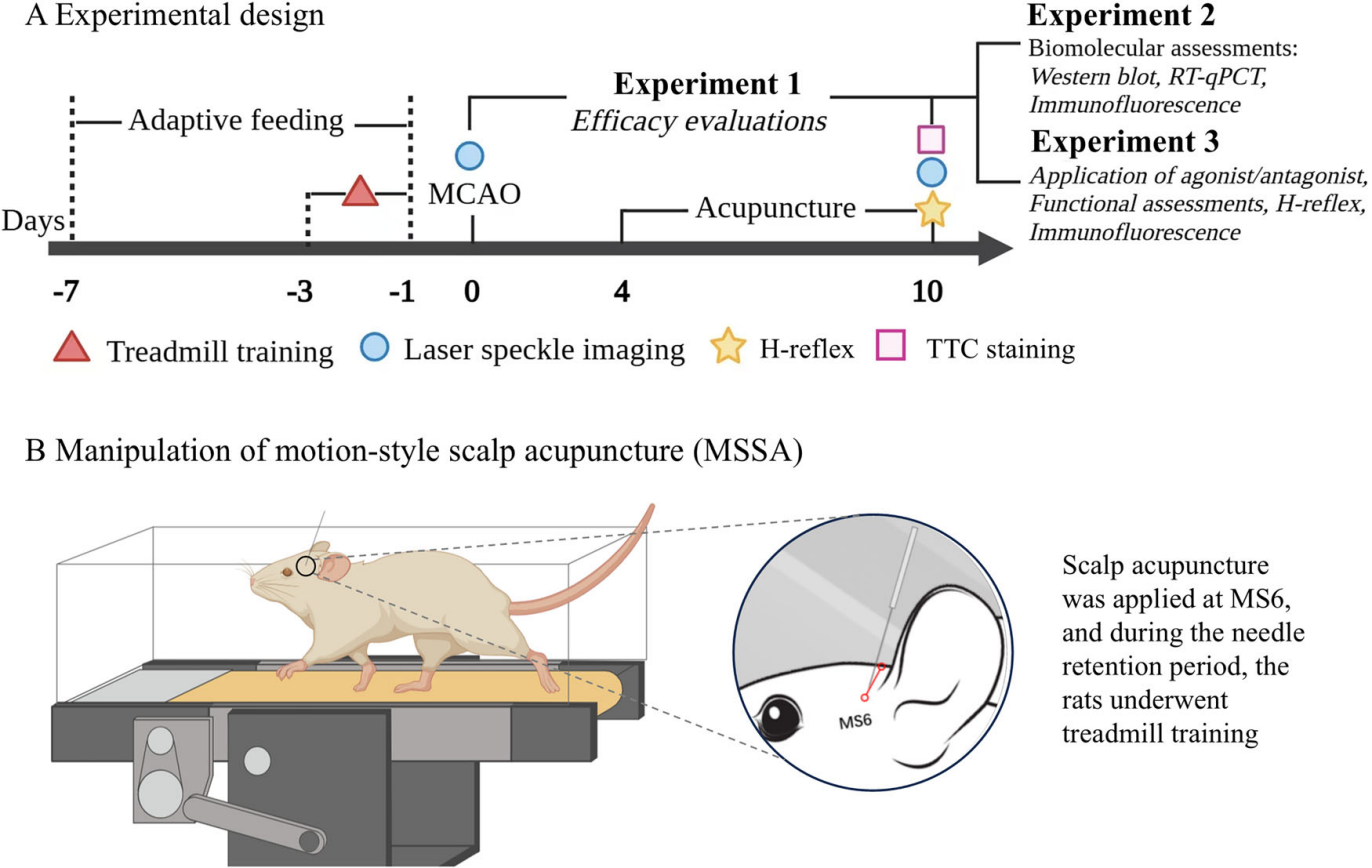
Schematic diagram of experimental design and treatment methods.

Treatment was initiated three days after MCAO surgery. The selected SA line was MS6 (anterior oblique line of the vertex‐temporal, extending from GV21‐*Qianding* obliquely to GB6‐*Xuanli*). In the MSSA group, a disposable acupuncture needle (ɸ 0.25 × 13 mm, Beijing Zhongyan Taihe Medical Instrument Co., Ltd) was transversely inserted to a depth of 10 mm at MS6 on the ischemic side. A rapid twirling manipulation (200 rpm) was performed for one min, with needle tails fixed by medical tape to prevent dislodgement, followed by needle retention for 30 min. During the needle retention period, the rats underwent treadmill training (10 m/min). A schematic diagram of MSSA is presented in Figure 1B. In the SA group, the acupuncture procedure was the same as that in the MSSA group, except that no treadmill training was involved. The treatment was given once a day and lasted for seven consecutive days. The rats in the sham‐operated and model groups did not receive any treatment but were subjected to the same fixation process as the treatment groups.

### 2.4 Drug Administration

The 5‐HT_2A_R agonist (5‐HT_2A_R agonist 2, HY‐162176, MCE, NJ, USA) and 5‐HT_2A_R antagonist (compound I14, HY‐151596, MCE) were individually dissolved in a solubilizing agent (serving as the vehicle control) composed of a mixture of 3/4 saline and 1/4 dimethyl sulfoxide (at a 1% concentration, HY‐10999A, MCE). In accordance with previous studies (Soares et al. 2024; Sun et al. 2022), rats were separately administered the 5‐HT_2A_R agonist, antagonist, or vehicle via gavage at a dosage of 0.125 mg/kg. Administration was carried out 20 mins prior to the acupuncture treatment from day 4 to day 10.

### 2.5 Experimental Design

#### 2.5.1 Experiment 1

To investigate the neuroprotective and antispastic effects of different acupuncture interventions. Seventy‐two rats were randomly allocated into four groups: the sham‐operated group (Sham), the model group (Model), the Model + MSSA group (MSSA), and the Model + SA group (SA). There were 20 rats in each group except for the Sham group (*n* = 12). The Zea Longa score, laser speckle imaging (LSI), and 2,3,5‐triphenyl tetrazolium chloride (TTC) staining were used to assess the changes in neurological deficit, rCBF, and cerebral infarction areas both before and after treatment. The MAS, H‐reflex recording, and screen test were used to evaluate the muscle tone, spinal reflex excitability, and motor function of the rats, respectively.

#### 2.5.2 Experiment 2

To explore whether 5‐HT_2A_R mediated spinal motoneuron KCC2 expression is implicated in the antispastic mechanisms of MSSA. The group allocation and treatment regimen were identical to those in Experiment 1. After the treatment, Western blot, RT‐qPCR, and double immunofluorescence labeling were used to assess the protein and gene expressions of 5‐HT_2A_R and KCC2.

#### 2.5.3 Experiment 3

To further elucidate the essential role of 5‐HT_2A_R in the antispastic effect of MSSA, the agonist and antagonist of 5‐HT_2A_R were administered. Sixty rats were randomly divided into five groups: Model, Model + 5‐HT_2A_R agonist 2, Model + vehicle, MSSA + compound I14, and MSSA + vehicle, with 12 rats per group. The Zea Longa score was utilized to assess the neurological deficit, the MAS and H‐reflex recording to evaluate the degree of muscle tone and excitability of spinal reflex, and double immunofluorescence labeling to observe the expression of 5‐HT_2A_R and KCC2 on the spinal motoneurons. The experimental design was presented in Figure 1A.

### 2.6 Behavioral Assessment

Behavioral assessments consisted of three components: the Zea Longa score for the evaluation of neurological deficit, the MAS for muscle tone, and the screen test for motor function. The Zea Longa and MAS were measured daily, commencing on the day prior to the MCAO surgery and continuing from 2 h after the surgery up to 10 days post‐surgery. The screen test was performed on day 3 (before treatment) and day 10 (after treatment).

#### 2.6.1 Zea Longa Score

The criteria of Zea Longa score are as follows: 0, no neurological deficit; 1, mild deficit that rats cannot fully extend left forepaw; 2, moderate deficit that body circling to the left; 3, moderate deficit that body falling to the left; 4, severe deficit that loss of ability to walk and consciousness. This indicates that the higher the score is, the worser the neurological deficits become (Longa et al. 1989).

#### 2.6.2 MAS

The MAS is used to assess muscle tone in the affected hind limb of rats, with a grading scale of 0 to 4. Higher scores indicate more severe spasticity: 0, no increase in muscle tone, with limb moving freely; 1, slight increase in muscle tone, with a catch and release or minimal resistance at the end of the range of motion (ROM) during flexion and extension; 1+, slight increase in muscle tone with a catch and minimal resistance throughout the remainder (less than half) of the ROM; 2, more marked increase in muscle tone through most of the ROM, with affected limb moving easily; 3, considerable increase in muscle tone, with difficulty in passive movement; 4, limited joint movement. The 1+ was converted into 2, so the maximum score of the MAS in this study was 5 (Meseguer‐Henarejos et al. 2018).

#### 2.6.3 Screen Test

The screen test device (Beijing Zhi Shu Duo Bao Biotechnology Co., Ltd, China) was made of 60 × 60 cm rough nylon wire with 1 × 1 cm areole. The net screen would automatically change from the horizontal to the vertical plane within 2 s and maintained in this position for another 5s. The amount of time that the rats were holding onto the net screen was calculated. The screen test is scored from 0 to 5, with lower grades indicating worse motor ability: 5, holding onto the screen and climbing upward freely; 4, holding onto the screen with forelimbs and not falling down within 5 s; 3, holding onto the screen temporally and slipping off a certain distance; 2, falling down onto the sponge within 5 s; 1, falling down onto the sponge immediately as soon as the screen was at the vertical plane (Wang et al. 2021).

### 2.7 Laser Speckle Imaging

The laser speckle imaging system (PeriCam PSI System, Stockholm, Sweden) was taken for measuring the rCBF in the MCA blood supply areas. The measuring times were settled at 5 min before and after the MCAO surgery, as well as after the treatment on day 10. Following anesthesia, the scalp of the rat was shaved, and an incision was made. The region of interest (ROI) was defined as a 2‐mm‐diameter area centered 2 mm posterior and 3 mm lateral to the bregma over the right cortices. The charge‐coupled device (CCD) camera was positioned 5 cm above the scalp, and images were captured with a 5‐ms exposure time and 11‐fps frame rate. The rCBF was quantitively expressed as the percentage in comparison with the pre‐MCAO baseline and analyzed using PIMsoft (Perimed AB, Stockholm, Sweden).

### 2.8 2,3,5‐TTC

After treatment, rats were deeply anesthetized by pentobarbital sodium (100 mg/kg, i.p.). The brain was harvested and frozen at –20°C for 30 min. Six coronal slices (2‐mm‐thick per slice) were dissected and then incubated in 0.3 % TTC solution (AMRESCO, USA) in the dark at 37°C. The slices were then fixed in 4 % paraformaldehyde solution and photographed. The percentage of brain slice infarction was quantified by ImageJ software (1.52q, NIH, USA) according to the following formula: Infarct area (%) = infract area/the whole brain slice area × 100 %.

### 2.9 H‐Reflex Recording

The spinal monosynaptic H‐reflex was recorded using the BL420N biological function experimental system recorder (Chengdu Taimeng Technology Co., Ltd., Chengdu, China). After anesthesia with isoflurane, the left sciatic nerve of the rat was bluntly dissected and hooked with a bipolar hook electrode to generate electrical stimuli. Oleo vitamin was dripped to protect the exposed nerve. A pair of stainless‐steel recording electrodes were inserted into the interosseus muscles of the left hind paw, and the ground electrode was inserted into the tail. The H‐reflex was then evoked by an isolated pulse stimulator delivering single bipolar pulses (100 µs). The activation threshold of the H‐reflex and the motor threshold (MT) could be determined by recording the H‐waves and M‐waves in response to a series of ascending stimulus intensities.

Furthermore, the frequency‐dependent depression (FDD) of the H‐reflex was observed (Mu et al. 2022). The same stimulus intensity that evoked the Hmax response was applied to a range of sequential stimulus pulses (at frequencies of 0.3, 5, and 10 Hz), and the amplitude changes of the H‐reflex under various stimulus frequencies were obtained. The 0.3 Hz pulses were repeated to confirm that the amplitude of the M‐wave remained within 95 % of the initial test; if not, the test was discarded. The calculation method for the FDD of the H‐reflex was based on the ratio of the H‐wave amplitude to the M‐wave amplitude (H/M ratio) at a stimulus frequency of 0.3 Hz. The percentages of the H/M ratio at 5 Hz and 10 Hz stimulus frequencies relative to that at 0.3 Hz were calculated, respectively.

### 2.10 Western Blot Analysis

Certain amounts of the spinal lumber enlargement (L4–L6 segments) were homogenized in radioimmunoprecipitation assay buffer containing protease inhibitors, after being removed from a ‐80°C freezer. The homogenate was ultrasonicated for 10 s using an ultrasonic disruptor, then centrifuged at 15,000 × g for 15 min at 4°C to collect the supernatant. Protein concentration was determined by BCA assay, and 25 µg of proteins were loaded onto a 10% SDS‐PAGE gel (with pre‐stained molecular weight markers) for electrophoresis and transferred to a polyvinylidene difluoride membrane (Millipore, MO, USA).The membranes were blocked with 5% BSA in TBST for 1 h and incubated overnight at 4°C with primary antibodies: 5‐HT_2A_R (Rabbit anti‐5‐HT_2A_R, 1:500, Bioss, Beijing, China), KCC2 (Mouse anti‐KCC2, 1:1000, Abcam, Cambridge, UK), and GAPDH (Mouse anti‐GAPDH, 1:1000, TA‐08, ZSGB‐BIO, Beijing, China), prior to incubation with secondary antibodies against goat anti‐rabbit IgG (H + L) HRP (1:1000, A0208, Beyotime) and goat anti‐mouse IgG (H + L) HRP (1:1000, 115‐035‐113, Jackson Immunoresearch, West Grove, PA, USA). Band intensities were quantified using ImageJ software, with relative expression normalized to GAPDH.

### 2.11 RT‐qPCR Analysis

The total RNA of the spinal lumbar enlargement was isolated with TRIZOL (Invitrogen, Carlsbad, CA, USA), and reversely transcribed into cDNA with Prime‐Script RT kit (CW2569, CWBIO, Beijing, China). All the procedures followed by the basic PCR reaction conditions. The gene expression levels of 5‐HT_2A_R and KCC2 were quantified by SYBR FAST qPCR Kit Master Mix (2×) (KK4601, KAPA Biosystems, MA, USA), and examined by StepOne plus Real‐Time PCR Detection System (ABI, CA, USA). The following primer gene sequences were used: 5‐HT_2A_R: CACCGACATGCCTCTCCATT (forward), GGACACTGCCATGATGACCA (reverse); KCC2: AGAATCCAGCCAACACTCGG (forward), AAGTTTTCCCACTCCGGCTT (reverse); GAPDH: TCATTGACCTCAACTACATGG (forward), TCGCTCCTGGAAGATGGTG (reverse). Expression levels of relative genes were calculated by the 2^−△△Ct^ method and normalized to those of GAPDH.

### 2.12 Double Immunofluorescence Labelling

Following the basic steps of immunofluorescence (transcardial perfusion fixation with 4% paraformaldehyde, sucrose gradient dehydration, embedding, sectioning at 8 mm thickness, permeabilization, and blocking), spinal cord sections were attached to glass slides. The sections were immersed in primary antibodies against 5‐HT_2A_R (Mouse anti‐5‐HT_2A_R, 1:500, SC‐166775, Santa Cruz Biotechnology, USA) and KCC2 (Rabbit anti‐KCC2, 1:500, ab259969, Abcam) at 4°C overnight. After three 5‐min PBS rinses, the sections were incubated with secondary antibodies (anti‐rabbit, 1:400, SC‐516176, Santa Cruz Biotechnology, green; and anti‐mouse, 1:400, ab150108, Abcam, red), in the dark for 1 h at 37°C. DAPI stained cell nuclei before coverslipping. Images were acquired via Z‐stack serial scanning using an Olympus FV3000 laser scanning confocal microscope, and mean fluorescence intensity was analyzed with ImageJ software.

### 2.13 Data Analysis

SPSS 20.0 (IBM, Armonk, New York, USA) was used for data analysis, and all measurements were presented as mean ± standard deviation. Significance was determined by one‐way analysis of variance (ANOVA) and two‐way repeated measures ANOVA, followed by Bonferroni or Tukey's post hoc tests. *p*‐value less than 0.05 (*p* < 0.05) was considered statistically significant.

## 3 Results

### 3.1 MSSA Improves Neurological Deficits, Promotes rCBF Restoration and Reduces Cerebral Infarction Areas

**FIGURE 2:**
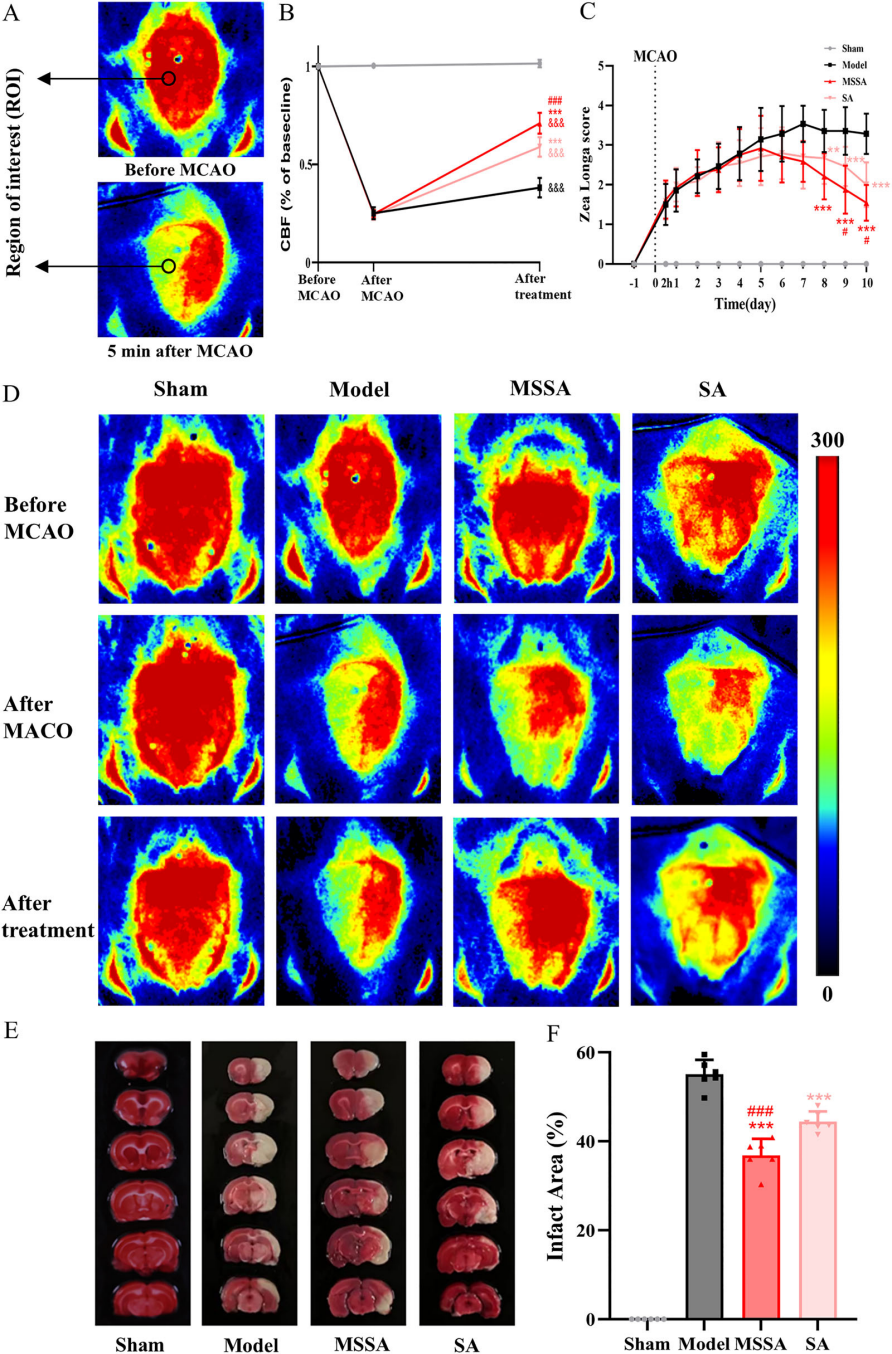
MSSA restored rCBF, reduced cerebral infarction areas, and improved neurological deficits in PSS rats. (A) Dynamic changes of rCBF were recorded in the ROI; (B) ROI values were quantified to evaluate changes of the rCBF at three time points, including pre MCAO, 5 min post MCAO and after intervention; (C) Zea Longa score was used for evaluating neurologic deficits of rats; (D) schematic diagrams of the rCBF changes in each group; (E) TTC staining of the rats brain; and (F) quantitative analysis of cerebral infarction areas. All the data were expressed as mean ± SD. And ^&&&^ *p* < 0.001 versus Sham group; ^***^ *p* < 0.001 versus Model group; ^###^ *p* < 0.001 versus SA group. MSSA, motion‐style scalp acupuncture; SA, scalp acupuncture.

The Zea Longa score was employed to evaluate the neurological deficits in rats. As shown in Figure 2C, all rats had a score of 0 on the day prior to MCAO. There were statistical differences over time (F[10, 26] = 13.672, *p* < 0.001), among groups (F[2, 35] = 9.683, *p* < 0.001), and in the interaction of these two factors (F[20, 52] = 2.383, *p* < 0.05). The Zea longa score in the Sham group remained constantly 0, whereas those in the modeling groups (Model, MSSA and SA groups) all increased, with no differences among the groups (all *p* > 0.05) from 2 h to the pre‐treatment period. These results signified that the MCAO surgery successfully induced neurological deficits in rats. Following seven consecutive days of treatment, the scores in the MSSA and SA groups gradually declined in comparison to those in the Model group (all *p* < 0.01, from day 8 to day 10). Notably, the scores in the MSSA group were significantly lower than those in the SA group (all *p* < 0.05, from day 9 to day 10).

Timely restoration of rCBF in the ischemic areas is reckoned with a promising approach for neuroprotection. Consequently, we used LSI to monitor the variation of rCBF 5 min before and after MCAO surgery, as well as after treatment. Regarding the rCBF, significant differences were detected over time (F[1,46] = 1152.659, *p* < 0.001], among groups (F[3, 46] = 1713.106, *p* < 0.001), and in the interaction of these two factors (F[3, 46] = 209.595, *p* < 0.001). As shown in Figure 2 A, B, and D, a pronounced decline (more than 70% reduction compared to the baseline) in rCBF was observed after MCAO in the Model, MSSA, and SA groups, indicating that the MCAO surgery effectively diminished the majority of the blood supply to the cortex centered around the ischemic core. Over the next 10 days, the rCBF tended to increase, with the MSSA and SA groups exhibiting obviously higher rCBF values than the Model group (all *p* < 0.001). Likewise, the recovery of rCBF in the MSSA group was superior to the SA group (*p* < 0.001).

Cerebral infarction areas were measured using TTC staining. As shown in Figure 2E and F, the brain slices in the Sham group were stained dark red; in contrast, those in the Model, MSSA, and SA groups displayed pale cerebral infarction and atrophy due to the inactivation of mitochondrial, which plays a crucial role in the process of ischemic neuronal apoptosis. After treatment, both MSSA (*p* < 0.001) and SA (*p* < 0.001) evidently reduced the infarction areas compared with the Model group. Besides, the reduction in infarction areas in the MSSA group was statistically greater than that in the SA group (*p* < 0.001). In summary, although both MSSA and SA contribute to the amelioration of neurological deficits and cerebral ischemia, the neuroprotective effects of MSSA are more superior to those of SA.

### 3.2 MSSA Alleviates Muscle Hypertonia, Enhances Motor Function and Inhibits Spinal Hyperreflexia

**FIGURE 3:**
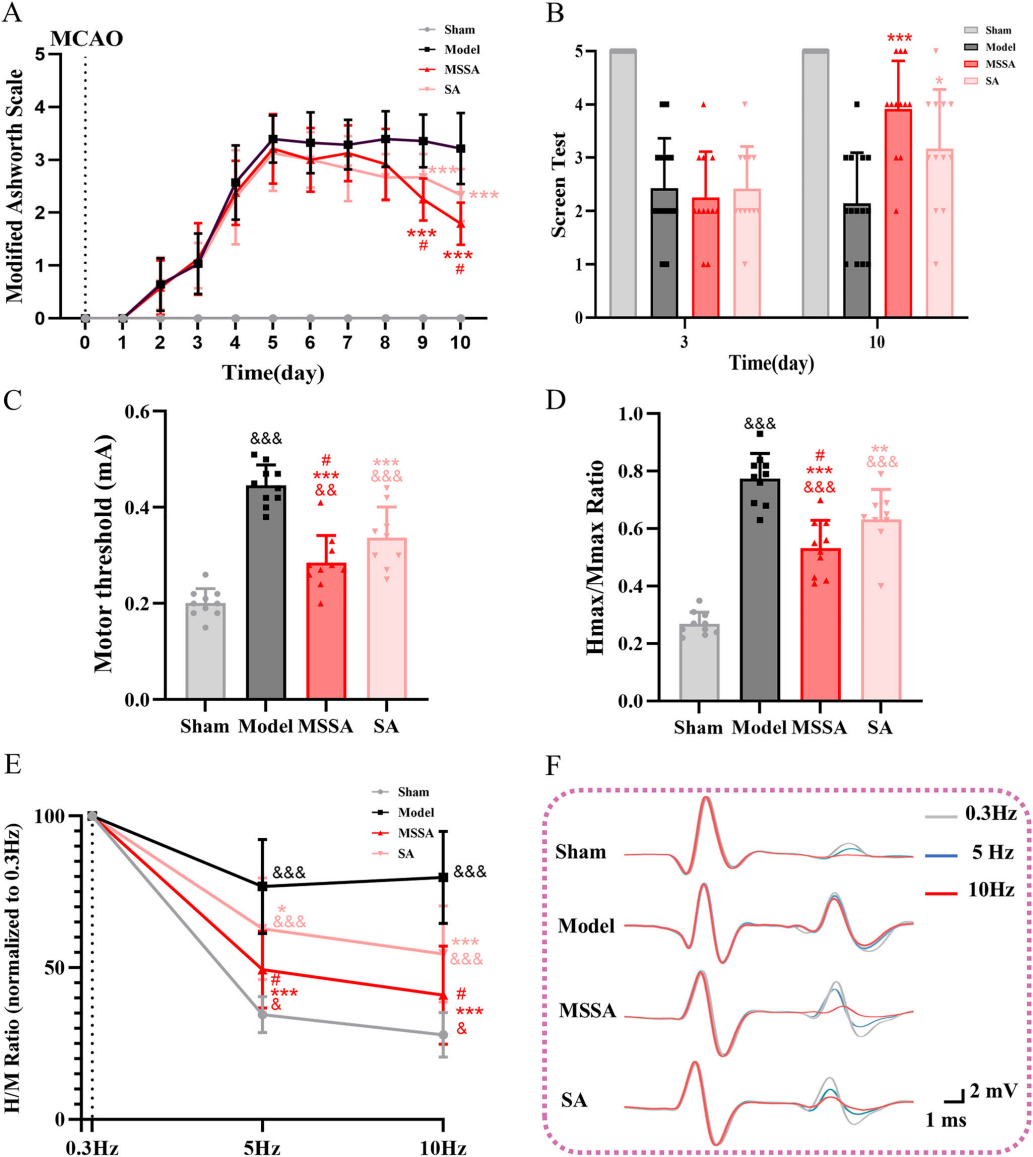
MSSA alleviated spastic muscle tone, motor dysfunctions and spinal hyperreflexia in PSS rats. (A) MAS for spastic muscle tone; (B) screen test for motor functions; (C) MT for the degree of spinal hyperreflexia; (D) Hmax/Mmax ratio was measured to estimate the proportion of motoneurons recruited by the monosynaptic reflex relative to the activation of the entire motor pool; (E) The FDD of the H‐reflex; (F) The representative H‐reflex recordings. The changes in H reflex after different stimuli at 0.3 Hz (light gray), 5 Hz (blue), and 10 Hz (red) were recorded once the FDD was impaired or not. All the data were expressed as mean ± SD. ^&^ *p* < 0.05, ^&&^ *p* < 0.01, ^&&&^ *p* < 0.001 versus Sham group; ^*^ *p* < 0.05, ^**^ *p* < 0.01, ^***^ *p* < 0.001 versus Model group; ^#^ *p* < 0.05 versus SA group. MSSA, motion‐style scalp acupuncture; SA, scalp acupuncture.

The MAS was performed to evaluate the muscle tone. In our study, the MAS score was consistently 0 in the Sham group, while the scores in the other model groups exceeded 1 at day 3, suggesting the occurrence of spastic hypertonia. Significant time (F[8,31] = 119.508, *p* < 0.001) and intervention effects (F[2, 35] = 10.430, *p* < 0.001) were observed in the MAS scores. The scores in the MSSA and SA groups were significantly lower than those in the Model group (all *p* < 0.001, at day 9 and 10). Moreover, in comparison with SA, MSSA effectively reduced the MAS scores (all *p* < 0.05, at day 9 and 10) (Figure 3A).

The screen test was used to comprehensively assess the motor ability of rats, including muscle tone, strength, stamina and balance (Wang et al. 2021). At day 3, the score was 5 in the Sham group but was obviously decreased in the Model, MSSA, and SA groups. After treatment, MSSA and SA effectively enhanced the motor ability of rats, with increased scores (F[2, 35] = 10.550, *p* < 0.001) compared to the Model group. No statistical differences were detected between the MSSA and SA groups (Figure 3B).

H‐reflex is frequently used to detect α‐motoneuron excitability and synaptic transmission in rats with spastic hypertonia following spinal cord injury and ischemic stroke (Wieters et al. 2022). Thus, the properties of H‐reflex were recorded herein to observe the effects of MSSA on spinal hyperreflexia and increased muscle tone. After stimuli of the sciatic nerve, two electromyography responses were obtained: the M‐wave, which is evoked by the direct activation of motor axons, and the H‐reflex, which is elicited by the activation of Ia synaptic afferents. The detailed properties of M‐wave and H‐reflex are shown in Table 1, and statistical differences were only observed for the MT (F[3, 35] = 42.900, all *p* < 0.001) and Hmax (F[3, 35] = 40.936, *p* < 0.001) among different groups. In addition, the Hmax/Mmax ratio reflects the activation rate of the α‐motoneuron pool in the anterior horn of the spinal cord and further denotes the degree of spinal hyperreflexia (Wieters et al. 2022). Compared with the Model group, MSSA and SA markedly decreased the Hmax/Mmax ratio and MT (all *p* < 0.001, Figure 3C and D). Similar to the MAS results, the reductions in the Hmax/Mmax ratio and MT in the MSSA group were more pronounced than those in the SA group (all *p* < 0.05).

**TABLE 1:** Properties of the M‐wave and H‐reflex including latency, amplitude, and threshold.

	Group	MT(mA)	Mmax (mV)	Hmax (mV)	M latency (msec)	H latency (msec)	H‐reflex threshold (× MT)	Hmax (× MT)
Experiment 2	Sham (*n* = 10)	0.201 ± 0.030	6.079 ± 0.646	1.625 ± 0.261	4.010 ± 0.404	9.920 ± 0.850	1.004 ± 0.179	1.618 ± 0.160
	Model (*n* = 10)	0.446 ± 0.422^&&&^	6.326 ± 0.767	4.850 ± 0.488^&&&^	4.110 ± 0.468	9.790 ± 0.300	1.005 ± 0.074	1.664 ± 0.147
	MSSA (*n* = 10)	0.285 ± 0.056^&&&***#^	6.091 ± 0.895	3.278 ± 0.897^&&&***#^	3.870 ± 0.241	9.010 ± 0.493	1.006 ± 0.151	1.656 ± 0.195
	SA (*n* = 9)	0.337 ± 0.064^&&&**^	6.316 ± 0.670	4.012 ± 0.864^&&&*^	4.044 ± 0.503	10.278 ± 0.512	0.963 ± 0.105	1.637 ± 0.168
Experiment 3	Model (*n* = 6)	0.418 ± 0.040	6.323 ± 0.709	5.156 ± 0.790	4.233 ± 0.301	8.467 ± 3.747	0.958 ± 0.087	1.741 ± 0.126
	Model + 5HT_2A_ agonist 2 (*n* = 6)	0.287 ± 0.060^###^	6.355 ± 0.629	2.331 ± 0.346^###^	4.200 ± 0.590	9.967 ± 0.712	0.968 ± 0.082	1.741 ± 0.142
	Model + vehicle (*n* = 6)	0.405 ± 0.049	6.403 ± 0.536	5.113 ± 0.339	4.167 ± 0.459	10.033 ± 0.692	0.922 ± 0.093	1.653 ± 0.094
	MSSA + Compound I14 (*n* = 6)	0.418 ± 0.041^***^	6.120 ± 0.734	5.043 ± 0.443^***^	4.200 ± 0.438	9.967 ± 0.653	0.958 ± 0.084	1.772 ± 0.113
	MSSA + vehicle (*n* = 6)	0.298 ± 0.055	6.521 ± 0.571	1.812 ± 0.198	4.167 ± 0.520	10.000 ± 0.645	1.022 ± 0.406	1.765 ± 0.158

*Note*: MT, motor threshold; MSSA, motion‐style scalp acupuncture; SA, scalp acupuncture. All the data were expressed as mean ± SD. In Experiment 2, ^&&&^ *p* < 0.001 versus Sham group; ^**^ *p* < 0.01, ^***^ *p* < 0.001 versus Model group; ^#^ *p* < 0.05 versus SA group. In Experiment 3, ^###^ *p* < 0.001 versus Model and Molde + vehicle groups; ^***^ *p* < 0.001 versus MSSA + vehicle group.

Typically, the amplitude of the H‐reflex diminishes as the stimulus frequency rises, resulting in a reduction in the H/M ratio, which is known as the FDD of the H‐reflex. Conversely, an increase in this ratio implies a higher excitability of α‐motoneurons, which is closely related to spinal hyperreflexia. To investigate whether MSSA improves the FDD, H‐reflexes were separately elicited at frequences of 0.3, 5, and 10 Hz on day 10. As shown in Figure 3E and F, as the stimulus frequency increases (from 0.3 to 10 Hz), the FDD in all groups presents a downward tendency in comparison to the baseline at 0.3 Hz. Under the same stimulus frequency (5 and 10 Hz), the FDD in the Model, MSSA, and SA groups was all higher than that in the Sham group (all *p* < 0.05). Of note, both MSSA (all *p* < 0.001) and SA (*p* < 0.05 at 5 Hz and *p* < 0.001 at 10 Hz) significantly reduced the FDD relative to the Model group; furthermore, statistical differences were also present between the MSSA and SA groups (all *p* < 0.05). Taken together, these results confirm that the therapeutic efficacy of MSSA exceeds that of SA in terms of improving muscle tone and motor ability, as well as modulating the spinal reflex.

### 3.3 MSSA Enhances the Expressions of 5‐HT_2A_R and KCC2 in the Spinal Lumber Enlargement

**FIGURE 4:**
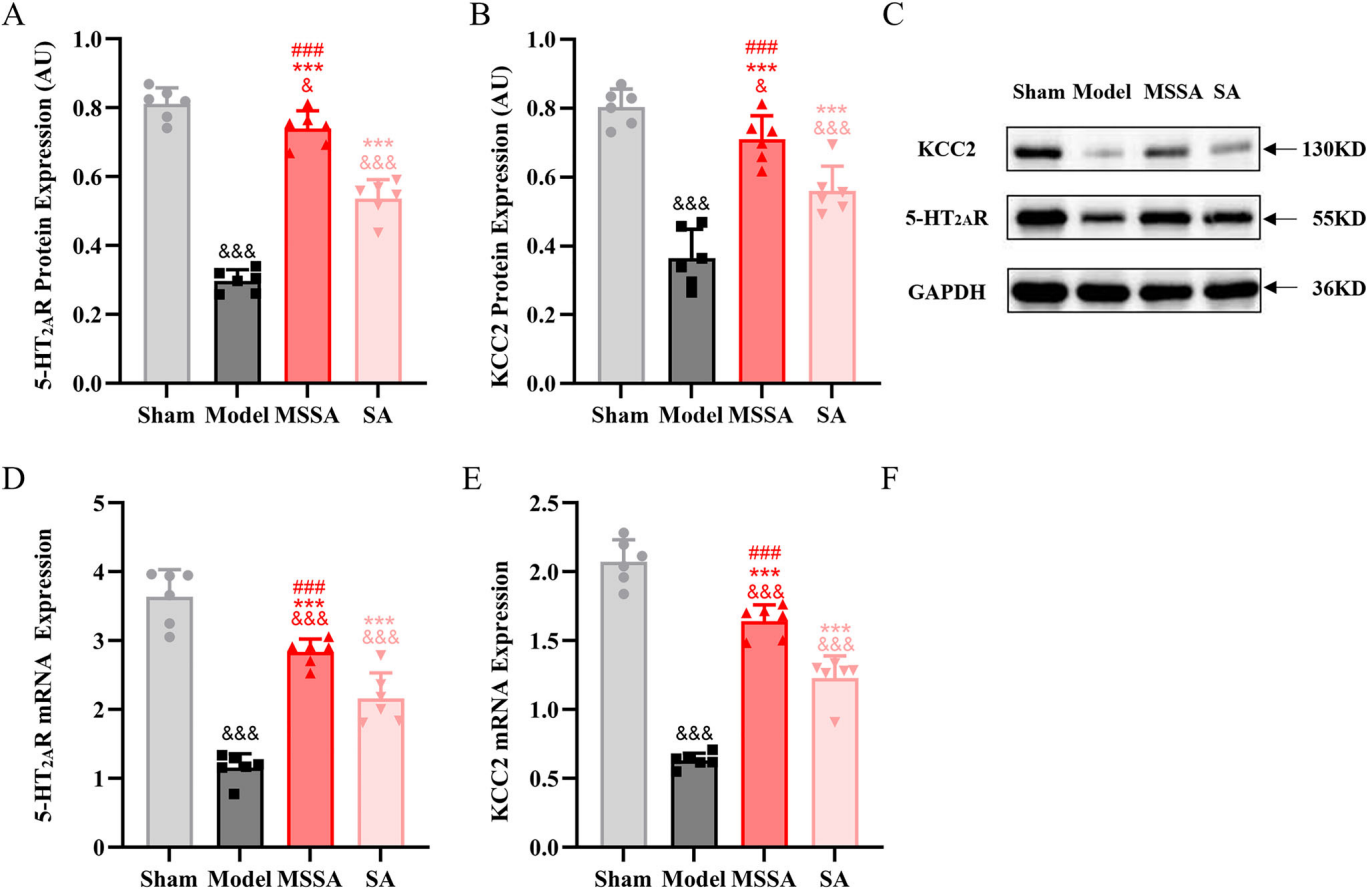
**MSSA elevated the expressions of 5‐HT_2A_R and KCC2 in the lumbar spinal cord**. (A, B) Western blot for the protein expressions of 5‐HT_2A_R and KCC2. (C) Representative western blot for 5‐HT_2A_R and KCC2 protein expressions relative to the GAPDH. (D, E) RT‐qPCR for the gene expressions of 5‐HT_2A_R and KCC2. All the data were expressed as mean ± SD. ^&^ *p* < 0.05, ^&&&^ *p* < 0.001 versus Sham group; ^***^ *p* < 0.001 versus Model group; ^###^ *p* < 0.001 versus SA group. MSSA, motion‐style scalp acupuncture; SA, scalp acupuncture.

Western blot and RT‐qPCR assays were conducted to examine the protein and mRNA expressions of 5‐HT_2A_R and KCC2. In terms of 5‐HT_2A_R, significant differences in the expressions of protein and mRNA were identified among the four groups (F[3, 20] = 145.270, *p* < 0.001; F[3, 20] = 71.639, *p* < 0.001, respectively). As depicted in Figure 4A and D, in comparison with the Sham group, the expressions of 5‐HT_2A_R protein and mRNA in the Model group were markedly reduced (all *p* < 0.001). After treatment, both MSSA (all *p* < 0.001) and SA (all *p* < 0.001) effectively augmented the expressions of 5‐HT_2A_R protein and mRNA relative to the Model group. Additionally, significant differences in the expressions of KCC2 protein and mRNA were observed among the groups (F[3, 20] = 45.350, *p* < 0.001; F[3, 20] = 133.029, *p* < 0.001, respectively) (Figure 4B and E). Similarly, in contrast to the Sham group, decreased expressions of KCC2 protein and mRNA were detected in the Model group (all *p* < 0.001); however, MSSA and SA effectively reversed the declined expressions of KCC2 protein and mRNA (all *p* < 0.001).

**FIGURE 5:**
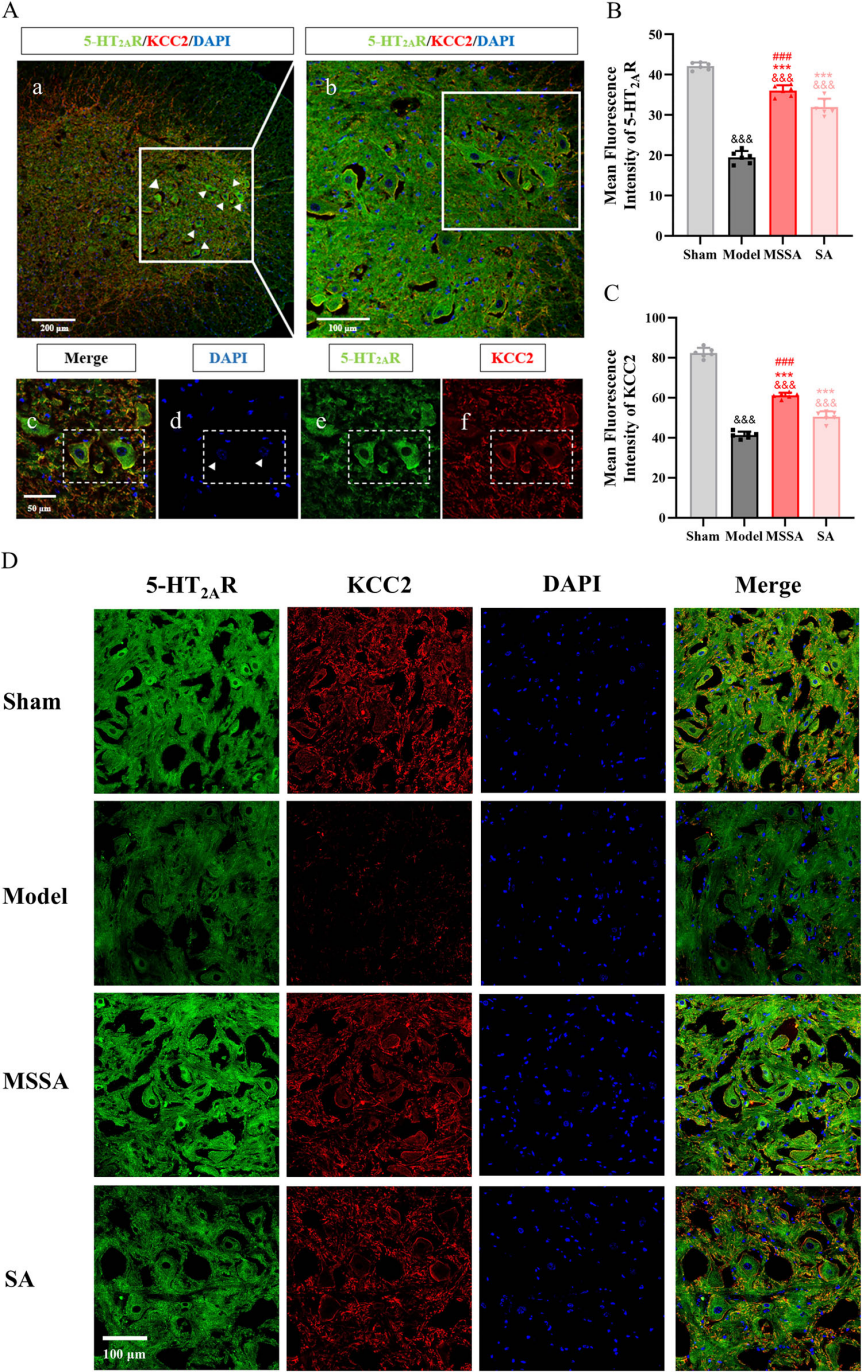
5‐HT_2A_R was co‐labeled with KCC2 in the motoneurons of the anterior horn of the lumber spinal cord. (A) The diagram showing the distribution of 5‐HT_2A_R (in green) and KCC2 (in red) within the spinal motoneurons, and the cell nuclei were stained with DAPI (in blue). Scale bar: (a) 200 µm; (b)100 µm; and (c–f) 50 µm. Mean fluorescence intensities of 5‐HT_2A_R (B) and KCC2 (C) were presented as the ratio of integrated intensity to the areas. (D) Immunofluorescence expressions of 5‐HT_2A_R and KCC2 in different groups, and the scale bar was 100 µm. All the data were expressed as mean ± SD. ^&&&^ *p* < 0.001 versus Sham group; ^***^ *p* < 0.001 versus Model group; ^###^ *p* < 0.001 versus SA group. MSSA, motion‐style scalp acupuncture; SA, scalp acupuncture.

Moreover, double immunofluorescence labeling was taken to investigate the coexistence and positional distribution of 5‐HT_2A_R and KCC2. Figure 5A–D presented the representative immunofluorescence images of 5‐HT_2A_R and KCC2 at different magnifications, which were highly expressed in the anterior horn of the spinal cord. Significant differences in the immunofluorescence expressions of 5‐HT_2A_R and KCC2 were noted among the groups (F[3, 20] = 229.261, *p* < 0.001; F[3, 20] = 409.302, *p* < 0.001, respectively). Compared with the Sham group, the expressions of 5‐HT_2A_R and KCC2 were evidently suppressed in the Model group (all *p* < 0.001). On the contrary, MSSA (all *p* < 0.001) and SA (all *p* < 0.001) were capable of enhancing the expressions of 5‐HT_2A_R and KCC2. More importantly, the enhancements of 5‐HT_2A_R and KCC2 expressions induced by MSSA were more pronounced than those by SA (all *p* < 0.001).

### 3.4 5‐HT_2A_R Antagonist Reverses MSSA Effects, While its Agonist Displays Similar Antispastic Efficacy to MSSA

**FIGURE 6:**
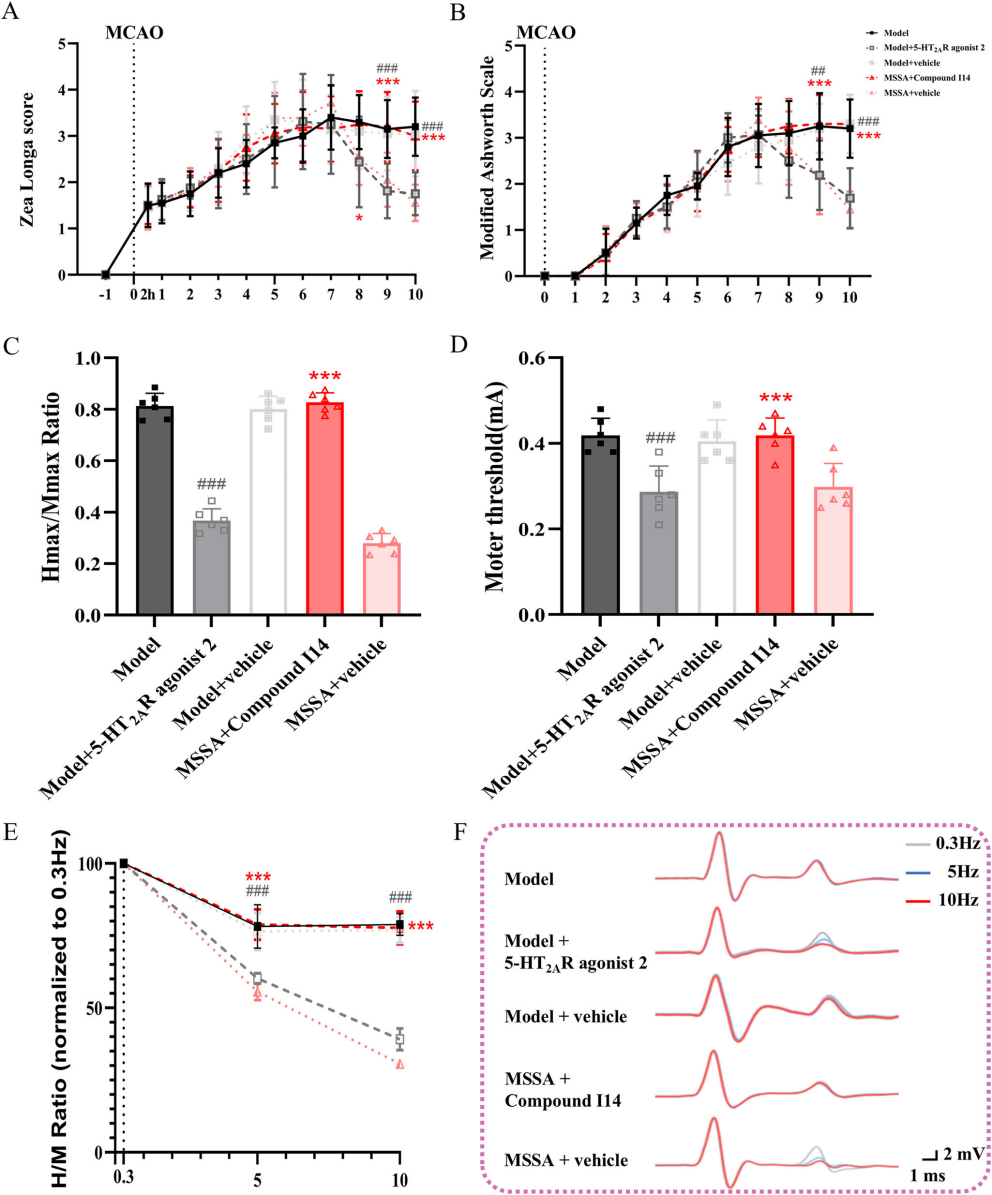
5‐HT_2A_R antagonist Compound I14 reversed therapeutic effects of MSSA, while its agonist exhibited similar effects to MSSA. (A) Zea Longa was utilized for assessing neurological deficits of rats; (B) MAS for spastic muscle tone; (C) Hmax/Mmax ratio; (D) MT; (E) the FDD of the H‐reflex; (F) representative H‐reflex recordings in different stimuli frequencies: 0.3 Hz (light gray), 5 Hz (blue), and 10 Hz (red). All data were expressed as mean ± SD. ^###^ *p* < 0.001 versus model and model + vehicle groups; ^***^ *p* < 0.001 versus MSSA + vehicle group. MSSA, motion‐style scalp acupuncture.

Based on the results of Experiment 1 and 2, preliminary inferences were made that the antispastic effects induced by MSSA were more pronounced than those of SA, and the underlying biomolecular mechanisms were associated with the augmented expressions of 5‐HT_2A_R and KCC2 in the spinal lumber enlargement. To further investigate whether these effects were contingent upon the activation of 5‐HT_2A_R, rats were administered Compound I14 and 5‐HT_2A_R agonist 2 separately and then subjected to relevant behavioral assays, electrophysiological recordings, as well as biomolecular detections. After treatment, the antispastic effects induced by MSSA were reversed by the administration of Compound I14. Specifically, the Zea Longa score, MAS score, MT, and Hmax/Mmax ratio in the MSSA + Compound I14 group were all significantly elevated, accompanied by a deteriorated FDD as manifested by an increased H/M ratio, when compared with the MSSA + vehicle group (all *p* < 0.001, Figure 6 A–E). In contrast to the Model group and Model + vehicle group, 5‐HT_2A_R agonist 2 effectively diminished the Zea Longa score, MSA score, MT, and Hmax/Mmax ratio, thereby facilitating the restoration of FDD (*p* < 0.001). Moreover, no statistical differences were found between the Model + 5‐HT_2A_R agonist 2 and MSSA + vehicle groups (all p > 0.05), suggesting that 5‐HT_2A_R agonist 2 mimicked the therapeutic effects of MSSA in ameliorating neurological deficits and spastic hypertonia.

**FIGURE 7:**
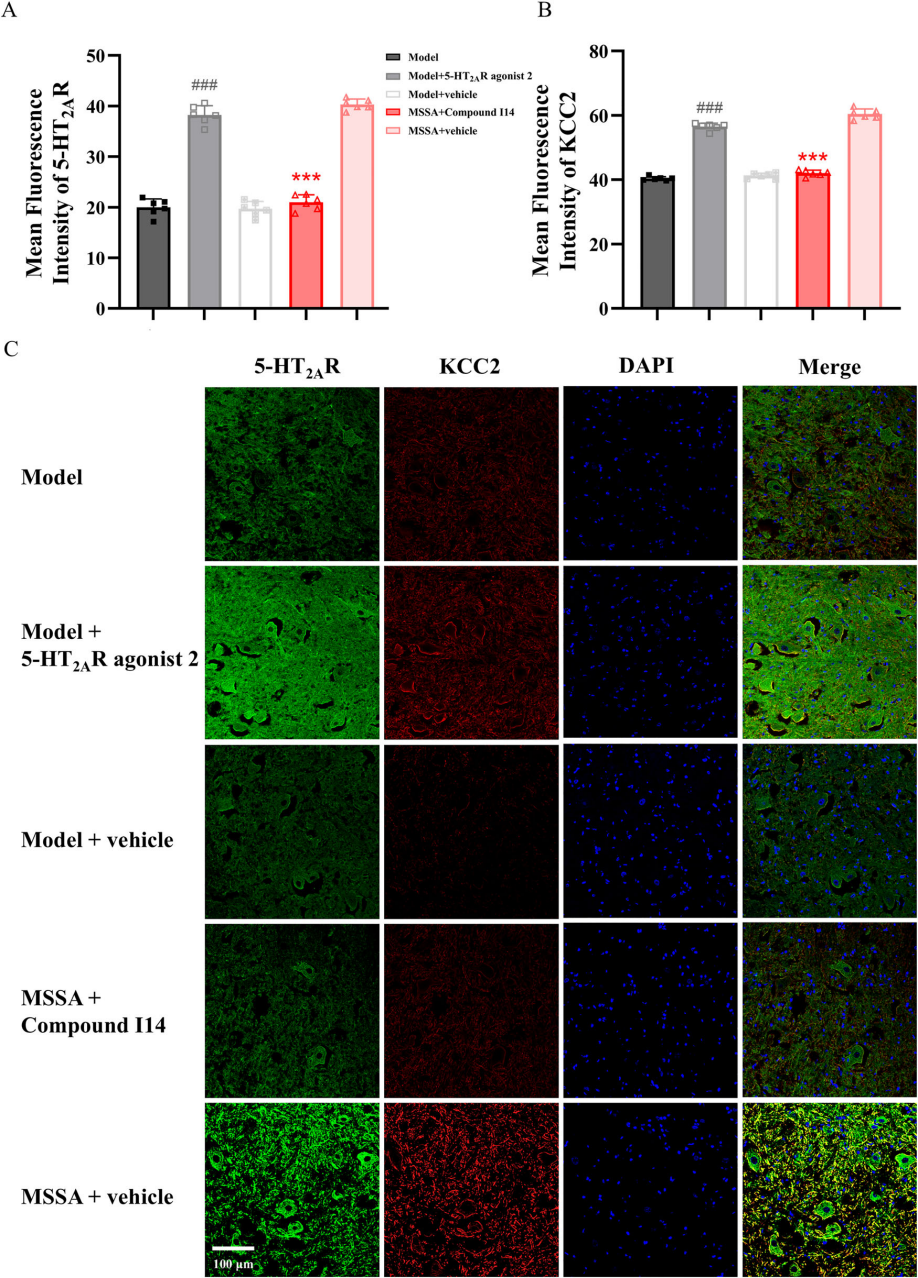
Influence of 5‐HT_2A_R agonist and antagonist on the expressions of 5‐HT_2A_R and KCC2 in the motoneurons of the lumber spinal cord. The mean fluorescence intensities of 5‐HT_2A_R (A) and KCC2 (B) were expressed as the ratio of the integrated intensity to the areas. (C) immunofluorescence expressions of 5‐HT_2A_R and KCC2 in different groups. Scale bar: 100 µm. All the data were expressed as mean ± SD. ^###^ *p* < 0.001 versus Model and Model + vehicle groups; ^***^ *p* < 0.001 versus MSSA + vehicle group. MSSA, motion‐style scalp acupuncture.

Furthermore, the mean fluorescence intensities of 5‐HT_2A_R and KCC2 were calculated. As presented in Figure 7 A–C, the expressions of 5‐HT_2A_R and KCC2 in the MSSA + Compound I14 group were declined relative to the MSSA + vehicle group (all *p* < 0.001). Conversely, the levels of these two proteins were significantly elevated in the Model + 5‐HT_2A_R agonist 2 group when compared with the Model + vehicle and Model groups (*p* < 0.001). These results further verified our hypothesis that 5‐HT_2A_R exerts a crucial role in the antispastic effect mediated by MSSA.

## 4 Discussion

The present study aimed to elucidate the biomolecular mechanisms underlying the therapeutic efficacy of MSSA in treating PSS. Our findings demonstrate that MSSA significantly improves neurological impairments and alleviates spasticity, outperforming traditional SA. The therapeutic effects of MSSA are mediated through multiple mechanisms: (1) enhancement of rCBF and reduction of cerebral infarction areas, which are critical for maintaining brain function and promoting recovery; (2) modulation of H‐reflex properties, a key indicator of neural excitability and reflex regulation; and (3) up regulation of 5‐HT_2A_R and KCC2 expression in the spinal lumbar enlargement. Notably, the 5‐HT_2A_R antagonist negated MSSA's therapeutic effects, while the 5‐HT_2A_R agonist mimicked them, supporting our hypothesis that MSSA's antispastic effects are partially mediated through 5‐HT_2A_R‐dependent KCC2 reactivation.

MSSA represents an innovative therapeutic approach that combines traditional SA with targeted exercise training, emphasizing individualized exercise regimens during needle retention. This integration has established MSSA as a specialized intervention for musculoskeletal disorders (Wang et al. 2023). SA, a modern acupuncture technique, aligns traditional methods with cerebral cortical functional zones (Jin et al. 2023). Studies have identified MS6, located above the cortical motor areas, as the most frequently used acupoint for post‐stroke motor impairments (Zhu et al. 2023). SA at MS6 promotes cerebral collateral circulation, enhances CBF, and facilitates gray matter remodeling in extrapyramidal motor centers, thereby improving motor function in stroke survivors (Jin et al. 2023; Lang et al. 2020). Additionally, early treadmill training has been shown to mitigate ischemic injury by reducing cerebral infarctions and suppressing neural apoptosis (Li et al. 2023; Sayyah et al. 2022). Interestingly, clinical studies consistently demonstrate that MSSA, combining SA and exercise training, yields superior outcomes in alleviating muscle spasticity and motor impairments compared to either intervention alone (Zhong et al. 2025). This study specifically explores the biomolecular mechanisms of MSSA at MS6 in PSS treatment.

Ischemic stroke progression involves irreversible damage to the ischemic core and detrimental changes in the salvageable penumbra (Paul et al. 2021). Reduced CBF in lesion‐dependent areas, such as the ipsilesional sensorimotor cortex, exacerbates muscle tone and functional impairments (Sztriha et al. 1996; Wiest et al. 2014; Wang et al. 2019). Ischemic insult volume is a significant predictor of PSS (Glaess‐Leistner et al. 2021), highlighting the importance of timely blood flow restoration to the penumbra for neuroprotection and subsequent neuroplasticity. In this study, LSI and TTC staining revealed that MSSA significantly improved rCBF and reduced cerebral infarction areas compared to SA and Model groups, corroborating its neuroprotective effects.

Post‐stroke motor recovery follows a sequential pattern, progressing from flaccidity to spasticity and eventually coordinated movement (Li et al. 2015). This process reflects maladaptive neuroplasticity, disrupting supraspinal‐spinal communication and leading to CST and lateral RST disinhibition, resulting in α‐motoneuron hyperexcitability (Wang et al. 2023; Li et al. 2015). The H‐reflex, a monosynaptic spinal reflex, serves as a reliable measure of motoneuron excitability and synaptic transmission (Misiaszek 2003). Increased Hmax/Mmax ratios and FDD alterations in PSS patients indicate spinal hyperreflexia (Shen et al. 2022; Thompson et al. 1992). In this study, MSSA demonstrated significant efficacy in normalizing H‐reflex parameters, as evidenced by reductions in MT, peak H‐wave amplitude, Hmax/Mmax ratio, and FDD of the H‐reflex. These electrophysiological improvements were corroborated by behavioral assessments using the MAS, collectively indicating that MSSA exerts superior antispastic effects compared to traditional SA.

In recent years, KCC2 has garnered significant attention for its pivotal role in spasticity recovery and motor function restoration (Talifu et al. 2022). Down regulation of KCC2 in spinal motoneuron membranes below the injury site depolarizes intracellular membrane potentials, representing a critical mechanism underlying spasticity development (Bilchak et al. 2021; Li et al. 2022). Our previous studies have demonstrated that acupuncture alleviates PSS by enhancing KCC2‐mediated GABAergic inhibition in the cerebral cortex, brainstem, and spinal cord (Wang et al. 2021; Mu et al. 2022; Sun et al. 2022). Furthermore, increased KCC2 activity in the spinal cord, coupled with improvements in FDD of the H‐reflex, has been shown to mitigate spasticity following spinal cord injury (Bilchak et al. 2021). These findings collectively underscore the therapeutic potential of interventions that enhance KCC2 expression and function for restoring endogenous inhibition and alleviating PSS.

Spinal motoneurons, the final output neurons for motor behaviors, are modulated by multiple neuromodulator systems, including serotonin (5‐HT) neurons (Kavanagh et al. 2022). Motoneuronal excitability is primarily regulated through 5‐HT2 receptor activation, particularly the 5‐HT_2A_R subtype (Thorstensen et al. 2024). Strong evidence indicates that 5‐HT_2A_R activation hyperpolarizes the reversal potential of inhibitory postsynaptic potentials (IPSPs) in spinal motoneurons by up regulating cell membrane KCC2 expression. This process enhances the function of stress‐impaired KCC2 and normalizes chloride ion homeostasis through protein kinase C (PKC)‐mediated signaling and phosphorylation of KCC2 at serine 940, which correlates with the alleviation of spasticity following spinal cord injury (Bos et al. 2013, Kimmey et al. 2019). In the present study, 5‐HT_2A_R and KCC2 expressions in the spinal cord were significantly reduced in the Model group, while MSSA effectively reversed these deficits. Notably, the 5‐HT_2A_R antagonist Compound I14 suppressed 5‐HT_2A_R expression, leading to decreased KCC2 levels in the spinal cord. This was accompanied by exacerbated behavioral deficits, as reflected by elevated Zea Longa and MAS scores, and impaired H‐reflex properties. Conversely, the 5‐HT_2A_R agonist mimicked the therapeutic effects of MSSA. These results further support our hypothesis that enhancing KCC2 function through 5‐HT_2A_R targeting in the spinal cord represents a promising therapeutic strategy for PSS.

### 4.1 Limitations and Future Prospects

This study has certain limitations. While the current research primarily focuses on comparing the antispastic efficacy and underlying mechanisms between MSSA and traditional SA, future investigations should incorporate comparisons between MSSA and exercise training, as well as explore the significance of combination timing. One underlying technical limitation remained: some images from the 5‐HT_2A_R immunofluorescence assay exhibit high background fluorescence and weak positive signals. Therefore, the results of this experiment can only be used as supplementary reference for western blot and PCR, and cannot independently support conclusions regarding 5‐HT_2A_R molecular expression. In future studies, the accuracy of 5‐HT_2A_R localization results could be further improved by optimizing antibody selection or adopting higher‐resolution spatial detection techniques such as in situ hybridization. Given that the diversity of PSS treatment regimens, future experiments should incorporate positive control groups receiving oral baclofen or botulinum toxin injection therapy. This inclusion will enable a more comprehensive clarification of the mechanistic advantages of MSSA compared to conventional spasticity management approaches.

## 5 Conclusion

In conclusion, our experimental findings demonstrate that MSSA exerts significant neuroprotective and antispastic effects in rats with PSS. These therapeutic outcomes are substantiated by measurable improvements in rCBF and notable modifications in H‐reflex properties. Mechanistically, these beneficial effects appear to be mediated through 5‐HT_2A_R‐regulated activation of spinal KCC2 expression. Furthermore, this study reinforces the critical influence of specialized acupuncture techniques on therapeutic efficacy, a principle that has gained substantial empirical support in clinical acupuncture practice.

## Author Contributions

**Jun‐Xiang Wang**: conceptualization, funding acquisition, methodology, supervision, writing – original draft, and writing – review and editing. **Liang‐Xiao Ma**: conceptualization, funding acquisition, writing – original draft, and supervision. **Qin‐Yong Zhang**: methodology, formal analysis, investigation, and data curation. **Jin‐Shan Zhong and Fei Li**: data curation and formal analysis. **Xu Qian, Ling‐Hui Ma, Jing‐Yun Xiu, and Xiu‐Yan Wang**: investigation. All authors read and approved the final manuscript.

## Funding

This research is supported by the National Natural Science Foundation of China (No. 82274655 and No. 82405579), the Fundamental Research Funds for the Central Universities of China (No. 2022‐JYB‐XJSJJ‐038), and the Scientific Research Development Fund Program of Beijing University of Chinese Medicine (No. ZJKT2023064).

Ethics Statement

All the procedures with animals were conducted in accordance with the Animal Ethics Committee of Beijing University of Chinese Medicine (Approval No. BUCM‐4‐2022070401‐3005).

## Conflicts of Interest

The authors declare no conflict of interests.

## Data Availability

The datasets generated and analyzed during the current study are available from the corresponding authors on reasonable request.
